# Comparison of total intravenous anesthesia and inhalation anesthesia on postoperative quality of recovery after laparoscopic hysterectomy: A protocol for systematic review and meta-analysis

**DOI:** 10.1097/MD.0000000000032365

**Published:** 2022-12-23

**Authors:** Menglin He, Mingxue Chen, Feng Yu

**Affiliations:** a Department of Anesthesiology, Shifang People’s Hospital, Sichuan, China; b Department of Anesthesiology and Pain, Nantong Haimen District People’s Hospital, Jiangsu, China.

**Keywords:** inhalation anesthesia, laparoscopic hysterectomy, meta-analysis, quality of recovery, total intravenous anesthesia

## Abstract

Postoperative recovery is a complex process and affected mainly by factors from patients, surgery and anesthesia. Although we have all kinds of sedatives and hypnotics now, the selection of an ideal medication for general anesthesia is still challenging. In this study, we perform a protocol for systematic review and meta-analysis to compare the effect of propofol-based total intravenous anesthesia and sevoflurane-based inhalation anesthesia on postoperative quality of recovery in patients undergoing laparoscopic hysterectomy.

**Methods:**

The protocol of this review was registered in PROSPERO (CRD42022379485). Meanwhile, it will be reported follow the guidelines of the Preferred Reporting Items for Systematic Reviews and Meta-Analyses Protocol. We will search 3 foreign electronic databases (Cochrane Library, Embase, Pubmed) and 4 Chinese electronic databases (China National Knowledge Infrastructure, WangFang Database, Chinese Biomedical Literature Database and Chinese Scientific Journal Database) to collect potential studies from their inceptions to December 2022. Only randomized controlled trials will be included. Two reviewers will independently perform study selection, data extraction and risk of bias assessment. Data synthesis and statistical analysis will be performed using the RevMan 5.4 (The Cochrane Collaboration, Copenhagen, Denmark) software.

**Results:**

The results of this systematic review and meta-analysis will be publicly available and published in a peer-reviewed journal.

**Conclusion:**

This study may provide the evidence regarding the efficacy and safety of total intravenous anesthesia and inhalation anesthesia on postoperative quality of recovery after laparoscopic hysterectomy.

## 1. Introduction

Laparoscopic hysterectomy, which was first described by Reich et al in 1989,^[1]^ is an excellent procedure in many cases where vaginal hysterectomy cannot be performed technically or when having other surgeries such as endometriosis or gynecological cancers associated at the same time. Compared to robotic hysterectomy, laparoscopic hysterectomy can be implemented in every hospital settings which has main operating theaters as it is more economical, accessible and sustainable.^[2,3]^ Although laparoscopic skills require a long and demanding learning curve, it is now an essential part of gynecology.^[4]^ Laparoscopic hysterectomy rates and trends have increased drastically in the last 2 decades compared to other hysterectomy types. The rates of laparoscopic hysterectomy, which accounts for only 1% of all hysterectomies in the 1990s, has reached 30% in many countries in recent years.^[5,6]^

Anaesthesia and surgery have certain inevitable negative impacts on the quality of life of patients, manifest as various discomforts after surgery even without specific complications.^[7]^ Moreover, prolonged recovery after surgery can lead to delayed hospital discharges and increased costs, which can impact resource utilization and mitigate patient satisfaction. The two most common general anesthesia techniques are total intravenous anesthesia (TIVA) and inhalation anesthesia.^[8]^ Most studies, however, have analyzed measures such as recovery time, cardiorespiratory perturbations, pain, nausea and vomiting, duration of the hospital stay, or other various adverse sequelae.^[9,10]^ Such piecemeal factors do not sufficiently reflect patient recovery from general anesthesia. A measurement that probes quality of life from the perspective of the patient is therefore an important factor in clinical studies that wish to investigate the effect of anesthesia and surgery on patient recovery and satisfaction. Therefore, we perform a protocol for systematic review and meta-analysis to compare the effect of propofol-based TIVA and sevoflurane-based inhalation anesthesia on postoperative quality of recovery in patients undergoing laparoscopic hysterectomy.

## 2. Methods

The protocol of this review was registered in PROSPERO (CRD42022379485). Meanwhile, it will be reported follow the guidelines of the Preferred Reporting Items for Systematic Reviews and Meta-Analyses Protocol.^[11]^ Ethical approval is not required because this review will retrieve publicly available scientific literature.

### 2.1. Inclusion and exclusion criteria

PICOS will be applied, including population, intervention, comparison, outcome, and study design.^[12]^

#### 2.1..1. Type of participants

Adults patients (age > 18 years) undergoing laparoscopic hysterectomy will be included. Regardless of gender, race, occupation, education, nationality, etiology, and severity.

#### 2.1..2. Type of intervention

Patients in intervention group receive propofol-based TIVA, regardless of the dose of administration used for anesthesia.

#### 2.1..3. Type of control

Patients in control group receive sevoflurane-based inhalation anesthesia.

#### 2.1..4. Type of outcome measurements

The primary outcome is quality of recovery-15 (QoR-15) questionnaire. We use the Chinese version of QoR-15, which has been validated as efficient and reliable as the original English version.^[13]^ It reflects the quality of postoperative functional recovery from 5 dimensions: physical comfort, emotional state, physical independence, psychological support and pain. The QoR-15 scores are recorded at 1 day before surgery, postoperative day 1 and postoperative day 3. Additional outcomes include length of hospitalization, operation time and adverse events.

#### 2.1..5. Type of study design

Only randomized controlled trials are included. The language will be limited to Chinese and English.

### 2.2. Search methods

We will search 3 foreign electronic databases (Cochrane Library, Embase, Pubmed) and 4 Chinese electronic databases (China National Knowledge Infrastructure, WangFang Database, Chinese Biomedical Literature Database and Chinese Scientific Journal Database) to collect potential studies from their inceptions to December 2022. The following search terms will be used: laparoscopic hysterectomy, intravenous anesthesia, inhalation anesthesia and quality of recovery. Search strategy for PubMed is shown in Table 1.

**Table 1:** Search strategy for PubMed.

#1 intravenous anesthesia [Title/Abstract]
#2 iv anesthesia [Title/Abstract]
#3 propofol [Title/Abstract]
#4 anesthetics intravenous [Title/Abstract]
#5 general anesthesia [Title/Abstract]
#6 TIVA [Title/Abstract]
#7 #1 OR #2 OR #3 OR #4 OR #5 OR #6
#8 inhalation anesthesia [Title/Abstract]
#9 volatile anesthesia [Title/Abstract]
#10 inhalational anesthesia [Title/Abstract]
#11 inhaled anesthesia [Title/Abstract]
#12 sevoflurane [Title/Abstract]
#13 #8 OR #9 OR #10 OR #11 OR #12
#14 laparoscopic hysterectomy [Title/Abstract]
#15 laparoscopic metrectomy [Title/Abstract]
#16 laparoscopic uterectomy [Title/Abstract]
#17 laparoscopically-assisted hysterectomy [Title/Abstract]
#18 #14 OR #15 OR #16 OR #17
#19 #7 AND #13 AND #18

TIVA = total intravenous anesthesia.

### 2.3. Selection of eligible studies

**Figure 1.**
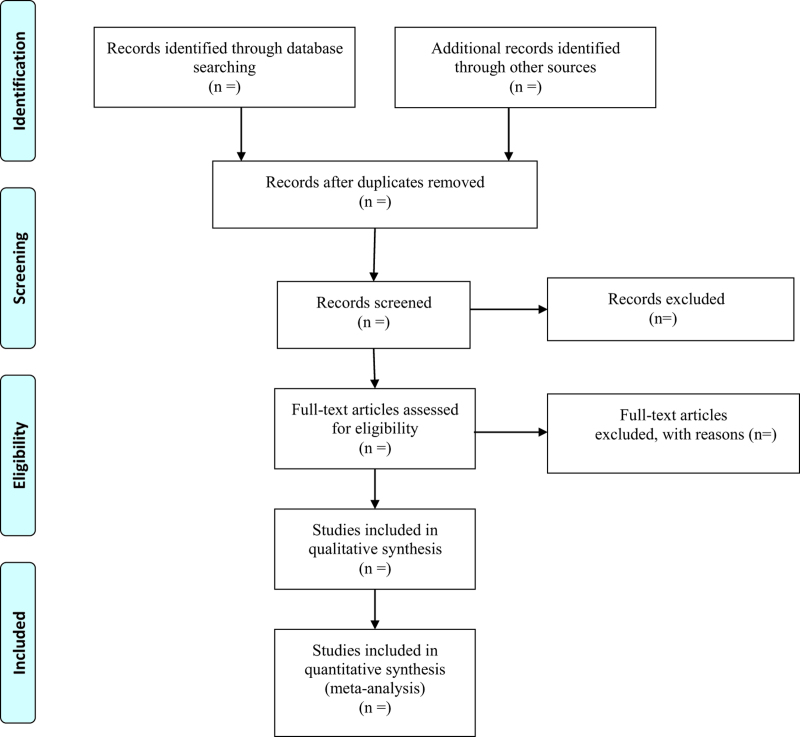
Flow diagram of study selection process.

Two reviewers will independently screen the titles and abstracts of the retrieved articles. We will also acquire the full text for screening to evaluate the eligibility for inclusion when necessary. Any disagreements will be resolved by discussion among reviewers. The process and results of the studies selection will be presented in a flow chart with Figure 1.

### 2.4. Data collection

The following data are extracted: author name; publication year; country of origin; study design; sample size; age; outcome measures and complications. Any differences of opinion will be resolved through group discussion or consultation with a third reviewer. When relevant data is not reported, we will contact the author via email or other means to obtain missing data.

### 2.5. Risk of bias assessment

The risk of bias will be assessed independently by 2 authors using the Cochrane tool of risk of bias.^[14]^ The following items will be assessed: random sequence generation (selection bias), allocation concealment (selection bias), blinding (performance bias and detection bias), incomplete outcome data (attrition bias), selective outcome reporting (reporting bias), and other bias. The judgments of evaluated domains will include high, low, and unclear. Disagreements will be resolved by discussion by arbiter.

### 2.6. Statistical analysis

Data synthesis and statistical analysis will be performed using the RevMan 5.4 (The Cochrane Collaboration, Copenhagen, Denmark) software. The standard mean difference with 95% confidence interval will be used to calculate the continuous data, while the dichotomous data will be measured by the rate ratio or odds ratio with 95% confidence interval. For the assessment of heterogeneity, the Chi-squared and *I*^2^ test will be carried out. If there is no significant heterogeneity among studies (*I*^2^ < 50%, *P* > .1), we will use a fixed-effect model, but a random-effects model will be employed if there exists heterogeneity (*I*^2^ ≥ 50%, *P* < .1).

### 2.7. Sensitivity analysis

Sensitivity analysis will be conducted by eliminating included studies one by one and changing the statistical methods to assess the stability and reliability of analytical results.

### 2.8. Assessment of reporting biases

We will conduct analysis of Egger publication bias plot and Begg funnel plot with pseudo 95% confidence limits to determine the publication bias in all the literature with sufficient studies.

### 2.9. Grading quality of evidence

We will use the Grading of Recommendations Assessment, Development, and Evaluation (GRADE) to assess the results.^[15]^ In the GRADE system, the quality of evidence will be categorized into 4 levels: high, moderate, low, and very low quality.

## 3. Discussion

Postoperative recovery is a complex process and affected mainly by factors from patients, surgery and anesthesia.^[16,17]^ Although we have all kinds of sedatives and hypnotics now, the selection of an ideal medication for general anesthesia is still challenging. Compared with objective examination data such as laboratory values and imaging, patients usually pay more attention to their self-perception of the disease and recovery. The QoR-15 is a recently developed patient-reported outcome measurement of postoperative quality of recovery.^[18]^ It was developed from the larger QoR-40,^[19]^ which has been extensively used and validated as a measurement of postoperative quality of recovery. The QoR-15 had equivalent psychometric properties compared with the QoR-40, but was more feasible to use. To our knowledge, this is the first meta-analysis to investigate the effect of propofol-based TIVA and sevoflurane-based inhalation anesthesia on postoperative quality of recovery in patients following laparoscopic hysterectomy. Further studies should focus on the optimal dose and anesthesia modality, as well as long-term adverse effects.

## Author contributions

**Conceptualization:** Mingxue Chen.

**Writing – original draft:** Menglin He.

**Writing – review & editing:** Feng Yu.
